# Clinical outcomes in patients receiving edoxaban or phenprocoumon for prevention of stroke in atrial fibrillation: a German real-world cohort study

**DOI:** 10.1186/s12959-022-00395-x

**Published:** 2022-07-04

**Authors:** Christopher Hohmann, Magnus Lutz, Sheila Vignali, Kathrin Borchert, Karolin Seidel, Sebastian Braun, Stephan Baldus, Michael Näbauer

**Affiliations:** 1 Department III for Internal Medicine, Heart Center, University of Cologne, Faculty of Medicine and University Hospital Cologne, Kerpener Str. 62, 50937 Cologne, Germany; 2 Daiichi Sankyo Deutschland GmbH, Zielstattstr. 48, 81379 Munich, Germany; 3 Xcenda GmbH, Lange Laube 31, 30159 Hannover, Germany; 4 Ludwig-Maximilians-University Munich, Marchioninistr. 15, 81377 Munich, Germany

**Keywords:** VKA, Edoxaban, Atrial fibrillation, Stroke, Claims data

## Abstract

**Background:**

Appropriate and timely anticoagulant therapy with vitamin K antagonists (VKAs) or non-vitamin K oral antagonists (NOACs) is essential for stroke prevention in non-valvular atrial fibrillation (NVAF). Comparative data regarding effectiveness and safety for edoxaban vs phenprocoumon, the predominant VKA in Germany, are scarce.

**Objectives:**

The study evaluates effectiveness and safety of edoxaban vs phenprocoumon in NVAF patients in a German real-world setting.

**Methods:**

German statutory health insurance claims data of the Institute for Applied Health Research Berlin (InGef) Research Database from 2014 until 2019 were analyzed. In NVAF patients, new users of edoxaban and phenprocoumon were compared to assess effectiveness (stroke/systemic embolism (SE)) and safety (bleeding) during therapy. Hazard ratios (HR) were estimated through multiple outcome-specific cox proportional hazard models adjusting for baseline characteristics. Outcomes of geriatric patients were analyzed in subgroup analyses.

**Results:**

Between 2015 and 2018, 7,975 and 13,319 NVAF patients newly initiated treatment with edoxaban or phenprocoumon. After adjusting for baseline confounders, the risk of stroke/SE (HR: 0.85, 95% CI: 0.70–1.02) was numerically but not significantly lower, while the risk of major bleeding (HR: 0.69, 95% CI: 0.58–0.81) was significantly lower for edoxaban. In the geriatric subgroups, homogenous results compared to the main analysis were obtained.

**Conclusion:**

The results of this real-world analysis indicated better effectiveness and safety outcomes in patients with NVAF initiating edoxaban treatment compared to phenprocoumon. The findings confirm that the beneficial effects observed in the pivotal ENGAGE AF-TMI 48 trial can also be achieved in real-world use of edoxaban.

**Supplementary Information:**

The online version contains supplementary material available at 10.1186/s12959-022-00395-x.

## Introduction

Non-valvular atrial fibrillation (NVAF) is the most prevalent cardiac arrhythmia and constitutes a major risk factor for stroke which results in increased mortality [1, 2]. The prevalence of atrial fibrillation (AF) in Germany ranges approximately between 2.1–2.5%, corresponding to 1.79 million affected individuals [3, 4]. Accordingly, appropriate and timely anticoagulant therapy of patients at risk with vitamin K antagonists (VKAs) or non-vitamin K oral anticoagulants (NOACs) is one of the core principles of AF management [5]. Despite high inter- and intrapersonal variation of exposure, multiple drug und food interactions, need of intensive International Normalized Ratio (INR)-monitoring, and risk of bleeding, VKAs have long been the standard of care for patients with NVAF [6]. Since June 19^th^, 2015, the direct factor Xa inhibitor edoxaban has been approved for stroke prevention in AF in Germany [7] after it has shown to be at least as effective and safer as the VKA warfarin in the pivotal ENGAGE AF-TMI 48 trial [8]. While the pivotal trial used warfarin as comparator, phenprocoumon is the predominant VKA in Germany. Therefore, comparative data on effectiveness and safety of edoxaban vs phenprocoumon need to be established [9].

This study was designed in analogy to the recent publication from Hohnloser et al. [10] in order to extend the research to the real-world effectiveness and safety profile of edoxaban compared to VKA phenprocoumon. The two treatment options were compared regarding effectiveness in prevention of stroke, systemic embolism (SE), all-cause mortality, and safety in terms of bleeding events.

Moreover, the frequent occurrence of geriatric characteristics such as high age, multi-morbidity, polypharmacy, and frailty in NVAF patients potentially influences effectiveness and safety of NOACs through several pathways such as treatment adherence, pharmacokinetics, drug interactions, and predisposition to side effects. Accordingly, different subgroup analyses of geriatric patients were performed following the framework laid out by Hohmann et al. [11].

## Methods

### Data source

The study was conducted as a non-interventional retrospective new-user cohort study using longitudinal German statutory health insurance (SHI) claims data of the Institute for Applied Health Research Berlin (InGef) Research Database. The research database comprises anonymized healthcare claims of more than four million covered lives insured in approximately 60 SHIs in Germany. This sample covers approximately 4.8% of the German population [12] and 5.6% of the German SHI population [13] as of 2020 and is structured to represent the German population in terms of age and gender according to the Federal Office of Statistics (DESTATIS [12]). The InGef Research Database was proven to have good external validity to the German population in terms of morbidity, mortality, and drug use [14]. Available data domains include core data regarding patients’ demographics, outpatient and inpatient healthcare services with diagnoses, procedures, and operations, prescription data, data on remedies, devices, and aids, and sick leave payments on an anonymized case-by-case level.

### Study timeframe

**Fig. 1:**
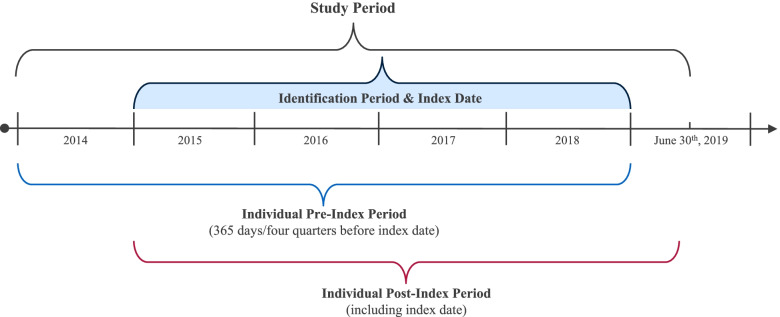
Study periods and timeframe

The study period spanned from January 1^st^, 2014 until June 30^th^, 2019. NVAF patients initiating treatment with edoxaban or phenprocoumon for stroke prevention were identified between January 1^st^, 2015 through December 31^st^, 2018. The index date was defined as the first edoxaban or phenprocoumon dispensation documented in the identification period and marked the beginning of the individual post-index period. Figure 1 gives an overview of the study periods and timeframes.

### Study population

The study population consisted of all NVAF patients who initiated edoxaban (60 mg or 30 mg) or phenprocoumon treatment between January 1^st^, 2015 and December 31^st^, 2018. All patients who had an ambulatory verified or primary or secondary hospital discharge diagnosis of AF in the previous or same quarter as the index date, who were aged ≥ 18 years in the index quarter, and who were continuously enrolled in the individual pre-index period of 365 days/four quarters before the index date were further included in the study.

Patients receiving any anticoagulant substance^1^ within the previous 365 days before the index date, more than one anticoagulant substance, or more than one dosage of edoxaban (60 mg and 30 mg) on the index date were excluded from the study. In addition, patients with at least one coded dialysis in the 365 days before or on the index date, patients receiving edoxaban/phenprocoumon and heparin on the index date, patients with documented cardiac valve surgery in the 365 days prior to the index date or on the index date, patients who presented any evidence of pregnancy in the four quarters prior to or in the index quarter, and patients with thrombosis or pulmonary embolism in the four quarters prior to or in the index quarter were excluded.

Study medication was identified by German Pharmaceutical Registration Numbers (PZN) or Anatomical Therapeutic Chemical Classification System (ATC) codes, diagnoses by outpatient and inpatient International Classification of Diseases, 10^th^ Revision, German Modification (ICD-10-GM) codes, and procedures based on Key of Operations and Procedures (OPS) for inpatient procedures and German Physician Fee Schedule (EBM) codes for outpatient procedures.

Baseline characteristics of the study population were descriptively assessed in the individual pre-index period of 365 days/four quarters preceding the index date and included demographic characteristics, healthcare resource utilization, comorbidities, concomitant medications, and risk scores (CHA_2_DS_2-_VASc^2^ [15], modified HAS-BLED^3^ [16], Charlson Comorbidity Index (CCI) [17–19]) associated with stroke and bleeding. If available, the definition and operationalization of covariates were set as closely aligned as possible with the publication of Hohnloser et al. [10] and the corresponding study report [20]. A definition of the respective baseline characteristics and the ICD-10-GM codes can be found in Additional file 1.

#### Geriatric patients

Subgroup analyses on geriatric patients were performed regarding age, comorbidities, polypharmacy, and frailty. In the first geriatric subgroup, the study population was stratified by age groups (< 65 years, 65–74 years, ≥ 75 years) with age determined in the index quarter.

For the second geriatric subgroup, comorbidities were assessed using the CCI weighing comorbidities that occurred in the pre-index period. A global CCI score for the total population of patients receiving edoxaban or phenprocoumon was calculated based on ambulatory verified as well as primary and secondary hospital discharge diagnoses within the 365 days/four quarters before the index date. Included conditions and their assigned weights were based on the original classification of diseases by Charlson et al. [17], incorporating the ICD-9 adaption by Deyo et al. [18] and ICD-10 adaption by Quan et al. [19]. High comorbidity was defined as scoring above the median score of the overall study population (individual CCI > median CCI).

The third geriatric subgroup was formed based on frailty, which was exploratorily applied according to a modified score based on auxiliary diagnoses from the publication of Segal et al. [21]. The approach of frailty classification using claims-based diagnoses was recently validated in the US against the Fried criteria as a gold standard of frailty assessment and showed a strong association with all-cause mortality and admissions to hospital or nursing homes [22]. To predict the probability of frailty for each patient in the study population, a claims-based frailty index (CFI) was calculated using the CFI variables and β coefficients from the adaptive lasso regression derived in Cardiovascular Health Study data by Segal et al. [21]. The median CFI of the overall study population was used to differentiate between frail (CFI > median CFI) and non-frail patients (CFI ≤ median CFI).

The fourth geriatric subgroup consisted of patients presenting with polypharmacy. Polypharmacy was defined as an intake of more pharmaceutical substances based on unique ATC codes on a 7-digit basis during the individual pre-index period of 365 days before the index date than the median of the overall study population (individual intake > median intake).

### Study outcomes

All study outcomes were assessed that occurred within the individual post-index period spanning from the index date until the occurrence of the first primary or secondary effectiveness or safety event investigated, the end of the study period on June 30^th^, 2019, the end of continuous enrollment (e.g., due to sickness fund switch or death), discontinuation of edoxaban or phenprocoumon treatment, or treatment switch to another anticoagulant therapy (including warfarin), whatever came first. The exposure start date for each patient was defined as the first edoxaban or phenprocoumon dispensation (dispense date) documented in the identification period spanning from January 1^st^, 2015 until December 31^st^, 2018. The exposure time was defined as the days of supply plus the days of not outcome-related hospitalization and a gap period of 30 days. As edoxaban is prescribed at a fixed dose, the number of days’ supply was set equal to the package size or the number of days until the new prescription. In order to account for the intra- and interpersonal variability of the phenprocoumon treatment regime (INR control and potential titration of phenprocoumon), an empirical defined daily dose based on the observed phenprocoumon prescription patterns was computed.

The primary effectiveness outcome of interest was the composite endpoint consisting of stroke (ischemic or hemorrhagic) and SE. Secondary effectiveness outcomes included all strokes, ischemic stroke, hemorrhagic stroke, and all-cause mortality. Effectiveness outcomes were identified based on primary or secondary hospital discharge ICD-10-GM diagnosis codes. A complete list of all ICD-10-GM codes used to identify the effectiveness endpoints and their operationalization is provided in Additional file 2. All-cause mortality included death from any cause.

The primary safety endpoint of interest was major bleeding and was defined as either cases with documented primary or secondary hospital discharge ICD-10-GM diagnosis codes of a major bleeding event in accordance with ICD-10-GM codes classified as major or intracranial bleeding, or hospital cases with an emergency admission in combination with an any bleeding or gastrointestinal bleeding event coded in accordance with ICD-10-GM codes classified as any or gastrointestinal bleeding and validated by the documentation of the OPS code 8-800 or the ICD-10-GM code D62. The secondary safety endpoints were intracranial bleeding, gastrointestinal bleeding, and any bleeding events, which were identified by primary or secondary hospital discharge ICD-10-GM diagnosis codes and OPS codes. A complete list of all ICD-10-GM and OPS codes used to identify the safety endpoints and their operationalization is provided in Additional file 3.

### Statistical analysis

For continuous variables such as age and number of hospitalizations, baseline characteristics were assessed using descriptive statistics including the number (n) and percentage (%) of subjects, mean, and standard deviation (SD). Frequencies and percentages were displayed for categorical data. Percentages by categories were based on the number of subjects with no missing data, i.e., added up to 100%. To estimate the balance between the treatment groups edoxaban vs phenprocoumon, the absolute standardized difference (ASD) was calculated. Phenprocoumon was used as the reference group while the threshold indicating imbalance was set to 0.1 [23].

Crude event rates and corresponding 95% confidence intervals (CIs) of the primary and secondary endpoints were described as the number of events per 100 person-years (% per year). Person-years of follow-up were calculated from the initiation of treatment with edoxaban or phenprocoumon to the occurrence of the first event investigated, the end of continuous enrollment, death, the end of the study period, discontinuation of treatment, or switching to another oral anticoagulant (including warfarin), whichever came first. Crude event rates for each endpoint and treatment group were calculated by dividing the number of events by the person time and reported per 100 person-years.

The adjusted event rates were calculated using a Poisson regression model, which considered a fictive patient who possessed the average baseline demographics and clinical characteristics of all patients. The primary and secondary endpoints served as dependent variables, while baseline characteristics and treatment groups were considered as independent covariates. Baseline variables were regarded time-independent, i.e., only the covariates’ value at baseline was considered. Adjusted event rates for the individual treatment groups were the marginal mean values of the prediction derived by the Poisson model and reported per 100 person-years.

To compare the risk for stroke/SE and all-cause mortality (effectiveness events), and bleedings (safety events) between NVAF patients initiating edoxaban and phenprocoumon treatment, hazard ratios (HR) were estimated through multiple outcome-specific cox proportional hazard regression models. Edoxaban was compared to phenprocoumon with phenprocoumon serving as reference category in the analysis. Adjustment included demographic characteristics, comorbidities, and risk factors for stroke/SE and bleeding as dependent variables, while the treatment groups were included as independent covariate. Covariates with significant influence on the multiple outcome-specific cox proportional hazard models were selected through machine learning in terms of backward elimination.

For building of the geriatric subgroups regarding age, comorbidity, frailty, and polypharmacy, the number and percentage of patients in each cohort and summary measures in terms of mean and SD were determined. Baseline characteristics and clinical features were stratified by treatment group (edoxaban vs phenprocoumon) and by subgroups of geriatric patients. In line with the main analysis, differences in the risk of primary and secondary effectiveness and safety events between the treatment groups were determined for geriatric patients. Multiple outcome- and geriatric subgroup-specific cox proportional hazard models were used to estimate the treatment effect on the respective event rates. Using analysis of variance, the effect modification by geriatric subgroups on the association between treatment and outcomes was tested by adding interaction terms for the age, comorbidity, frailty, and polypharmacy subgroups to the multiple cox proportional hazard regressions.

## Results

### Patient population

**Fig. 2:**
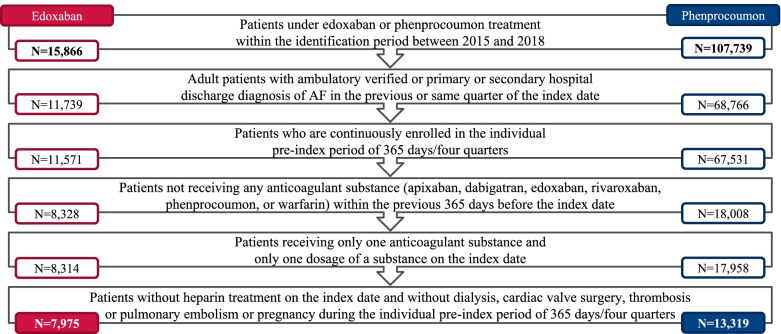
Flowchart of patient selection

Between January 1^st^, 2015 and December 31^st^, 2018, a total of 7,975 and 13,319 NVAF patients were identified who had newly initiated treatment with edoxaban or phenprocoumon and met all inclusion criteria. Most patients were excluded due to anticoagulant therapy within the 365 days before the index date (edoxaban: ~ 3,250 patients, phenprocoumon: ~ 49,500 patients). Compared to the overall population of patients with an edoxaban or phenprocoumon prescription, approximately 50% of edoxaban patients and 12% of phenprocoumon patients remained in the final study population (Fig. 2).

The results on baseline characteristics of the identified study populations showed that patients initiated on phenprocoumon were older compared to those initiated edoxaban (mean age 76.6 vs 73.7 years), were less likely to be male (52.5% vs 55.5%), had a higher healthcare resource utilization in terms of all-cause hospitalizations and hospital days, had an increased baseline risk of stroke (CHA_2_DS_2_-VASc score) and bleeding (modified HAS-BLED score), and a higher comorbidity burden. With regard to concomitant medication, no substantial differences were found between the groups with respect to antiplatelet drugs, acetylsalicylic acid (ASA), non-steroidal anti-inflammatory drugs (NSAIDs), ß-blockers, and proton-pump inhibitors (Table 1).

**Table 1:** Baseline characteristics

Characteristic	Edoxaban (*n* = 7,975)	Phenprocoumon (*n* = 13,319)	ASD^a^
**Demographics**			
Age (mean ± SD)	73.70 [10.81]	76.56 [8.90]	0.30
Male	55.5%	52.5%	0.06
**Health Resource Utilization**			
Number of all-cause hospitalizations (mean ± SD)	0.97 [1.20]	1.11 [1.33]	0.11
Number of hospital days (mean ± SD)	7.92 [15.94]	10.86 [18.10]	0.17
Number of all-cause hospitalizations within 30 days prior first dispensation (mean ± SD)	0.46 [0.56]	0.42 [0.57]	0.07
Number of stroke/SE-related hospitalizations within 30 days prior first dispensation (mean ± SD)	0.03 [0.17]	0.03 [0.18]	0.02
Number of outpatient cases (mean ± SD)	13.14 [7.33]	14.29 [7.59]	0.15
Number of unique medications (mean ± SD)	8.61 [5.08]	9.86 [5.25]	0.24
**Risk Scores**			
CHA_2_DS_2_-VASc (mean ± SD)	3.56 [1.64]	4.15 [1.52]	0.38
Modified HAS-BLED (mean ± SD)	2.52 [1.15]	2.82 [1.06]	0.27
Charlson Comorbidity Index (mean ± SD)	2.91 [2.59]	3.67 [2.72]	0.28
**Comorbidities**			
Coronary heart disease	33.6%	47.6%	0.29
Congestive heart failure	28.9%	41.7%	0.27
Renal insufficiency	18.4%	28.7%	0.24
Diabetes mellitus	30.3%	37.3%	0.15
Hypertension	83.3%	89.4%	0.18
Ischemic stroke or transient ischemic attack	9.6%	12.0%	0.08
Myocardial infarction	4.4%	8.3%	0.16
Major bleeding	1.0%	1.7%	0.06
Any bleeding event	6.7%	10.4%	0.13
**Concomitant Medication**			
Antiplatelet drugs	21.8%	24.3%	0.06
ASA	17.8%	18.5%	0.02
NSAIDs	34.2%	31.7%	0.05
ß-blockers	78.8%	82.0%	0.08
Proton-pump inhibitors	39.5%	44.9%	0.11

*Abbreviations*: *ASA* acetylsalicylic acid, *ASD* absolute standardized difference, *NSAIDs* nonsteroidal anti-inflammatory drugs, *SD* standard deviation, *SE* systemic embolism

^a^ ASD > 0.1: An absolute value greater than 0.1 was defined as indicating imbalance between edoxaban vs phenprocoumon

### Effectiveness outcomes

The number of events and corresponding crude and adjusted event rates per 100 person-years for the effectiveness outcomes are listed in Table 2 stratified by edoxaban and phenprocoumon treatment. Overall, there were 579 patients with stroke/SE during follow-up. Among these, 385 patients experienced an ischemic stroke, and 119 patients suffered a hemorrhagic stroke, respectively. Unadjusted (crude) event rates were slightly higher for stroke/SE, all strokes, hemorrhagic strokes, and all-cause mortality for phenprocoumon in comparison to edoxaban. Ischemic stroke events were slightly more frequent in patients treated with edoxaban.

**Table 2:** Crude and adjusted event rates of effectiveness and safety outcomes for edoxaban vs phenprocoumon

Outcome		Edoxaban				Phenprocoumon			
		Patients with events	Person-years of follow-up	Crude rate per 100 person-years	Adjusted rate per 100 person-years	Patients with events	Person-years of follow-up	Crude rate per 100 person-years	Adjusted rate per 100 person-years
**Effectiveness**	Stroke/SE	158	8,101	2.0	1.7	421	19,323	2.2	1.8
	All strokes	138	8,108	1.7	1.5	356	19,373	1.8	1.6
	Ischemic stroke	119	8,111	1.5	1.3	266	19,388	1.4	1.2
	Hemorrhagic stroke	23	8,157	0.3	0.2	96	19,491	0.5	0.4
	All-cause mortality	561	8,161	6.9	4.5	1,477	19,506	7.6	4.4
**Safety**	Major bleeding	191	8,105	2.4	2.1	696	19,172	3.6	2.9
	Intracranial bleeding	43	8,152	0.5	0.5	206	19,445	1.1	0.9
	Gastrointestinal bleeding	254	8,056	3.2	2.8	567	19,127	3.0	2.3
	Any bleeding	837	7,801	10.7	10.5	2,547	17,666	14.4	12.4

*Abbreviations*: *SE* systemic embolism

**Fig. 3:**
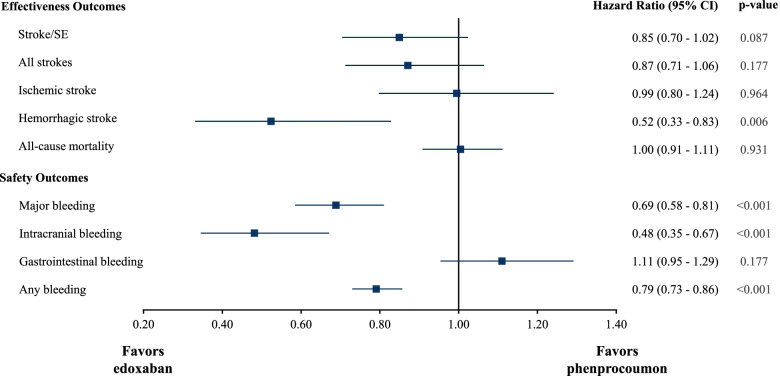
Hazard ratios and 95% CI for effectiveness and safety outcomes

After adjusting for baseline confounders, edoxaban was associated with significantly lower risk for hemorrhagic stroke than phenprocoumon (HR: 0.52, 95% CI: 0.33–0.83). Furthermore, edoxaban showed a numerically lower risk of stroke/SE (HR: 0.85, 95% CI: 0.70–1.02) and all strokes (HR: 0.87, 95% CI: 0.71–1.06) compared to phenprocoumon without reaching statistical significance, while the risks of ischemic stroke (HR: 0.99, 95% CI: 0.80–1.24) and all-cause mortality (HR: 1.00, 95% CI: 0.91–1.11) were similar between the treatment groups. HRs and respective 95% CIs and *p*-values for the effectiveness outcomes are displayed in Fig. 3.

### Safety outcomes

The number of safety events and corresponding crude and adjusted event rates per 100 person-years stratified by edoxaban and phenprocoumon treatment are displayed in Table 2. During follow-up, a total of 3,384 patients experienced any bleeding event at any localization. Thereof, 887 patients suffered from a major bleeding, while another 249 patients had an intracranial bleeding and 821 patients a gastrointestinal bleeding. The comparison of unadjusted event rates indicated lower crude event rates of major bleeding, intracranial bleeding, and any bleeding in patients treated with edoxaban compared to patients treated with phenprocoumon, while gastrointestinal bleedings were more prominent in patients initiating edoxaban treatment.

The adjusted HRs for the safety outcomes revealed that edoxaban was associated with significantly lower risks for major bleeding (HR: 0.69, 95% CI: 0.58–0.81), intracranial bleeding (HR: 0.48, 95% CI: 0.35–0.67), and any bleedings (HR: 0.79, 95% CI: 0.73–0.86) in comparison to phenprocoumon. The risk for gastrointestinal bleeding was numerically but not significantly higher for edoxaban (HR: 1.11, 95% CI: 0.95–1.29). The HRs and respective 95% CIs and *p*-values for the safety outcomes are displayed in Fig. 3.

### Subgroups of geriatric patients

The subgroup analyses of geriatric patients revealed that patients initiated on phenprocoumon were older (65.5% vs 53.7% ≥ 75 years at treatment initiation), had a higher comorbidity burden (46.2% vs 33.8% with high CCI), were rather classified as frail (53.5% vs 44.1% with frailty), and presented more frequently with polypharmacy (46.8% vs 36.5% with polypharmacy) compared to patients starting edoxaban treatment (Table 3). In the baseline period, geriatric characteristics and treatment with phenprocoumon were associated with increased healthcare resource utilization and comorbidity burden as well as higher risk scores and increased use of concomitant medications.

**Table 3:** Geriatric subgroups

Geriatric Subgroups		Edoxaban		Phenprocoumon	
		**n**	**%**	**n**	**%**
**Age**	< 65 years	1,454	18.2%	1,306	9.8%
	65–74 years	2,238	28.1%	3,295	24.7%
	≥ 75 years	4,283	53.7%	8,718	65.5%
**Comorbidity**	High CCI score	2,698	33.8%	6,156	46.2%
	Low CCI score	5,277	66.2%	7,163	53.8%
**Frailty**	Frail	3,513	44.1%	7,131	53.5%
	Not frail	4,462	55.9%	6,188	46.5%
**Polypharmacy**	Polypharmacy	2,912	36.5%	6,231	46.8%
	No polypharmacy	5,063	63.5%	7,088	53.2%

*Abbreviation*: *CCI* Charlson Comorbidity Index

**Fig. 4:**
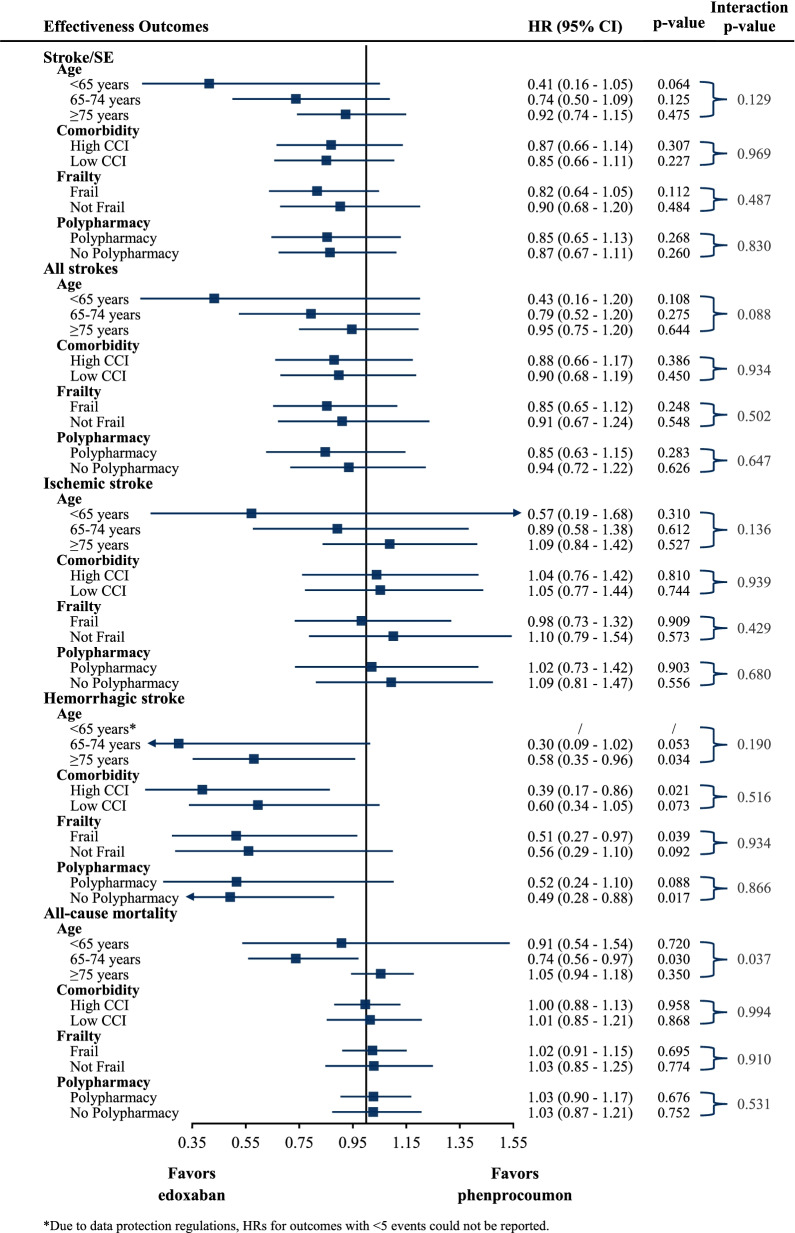
Hazard ratios and 95% CI for effectiveness outcomes in geriatric subgroups

**Fig. 5:**
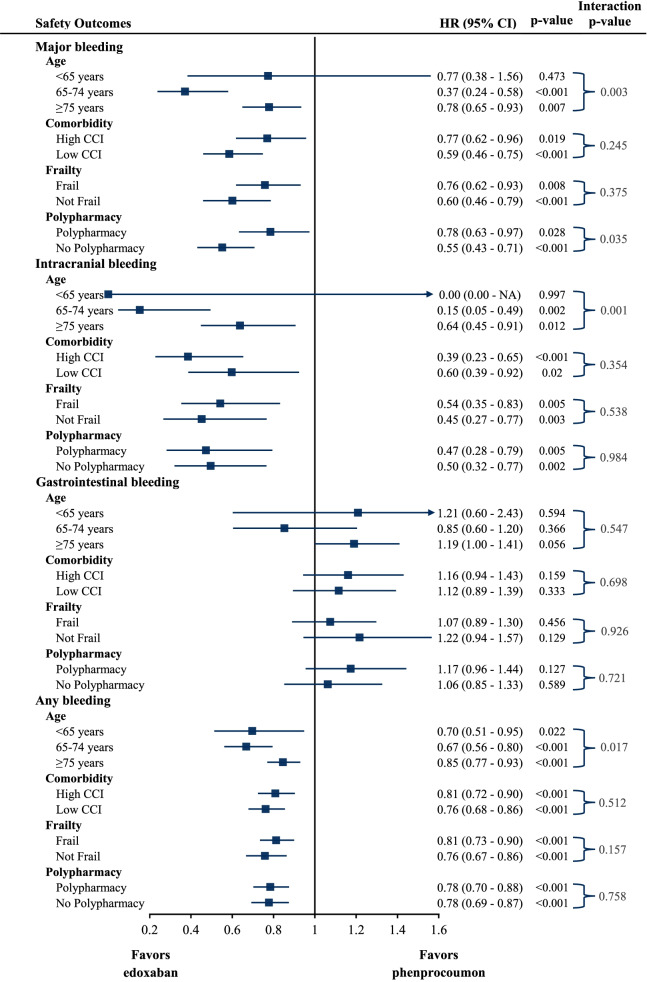
Hazard ratios and 95% CI for safety outcomes in geriatric subgroups

The analysis of the effect modification by geriatric subgroups on the associations between treatment and outcomes revealed a similar risk for the occurrence of an effectiveness or safety outcome on edoxaban treatment compared to phenprocoumon with respect to age, comorbidity burden, frailty level, and presence of polypharmacy when considering the adjusted HRs. The treatment effect was not significantly different for effectiveness outcomes by any geriatric characteristic despite of all-cause mortality in age groups. For safety outcomes, a significantly different treatment effect was observed regarding major bleeding between age and polypharmacy subgroups and for intracranial and any bleeding between age subgroups. Overall, the subgroup analyses yielded homogenous results and effects pointed in the same direction as in the main analysis, with effect modification only occasionally observed (Figs. 4 and 5).

## Discussion

The present study compared the effectiveness and safety profile of edoxaban to that of phenprocoumon in patients with NVAF in Germany initiating treatment between 2015 and 2018. The results of this real-world analysis indicate better effectiveness and safety outcomes in patients initiating edoxaban treatment compared to phenprocoumon. The findings are largely consistent with the results of comparable real-world studies [9, 10] and confirm the applicability of the results of the pivotal ENGAGE AF-TIMI 48 trial [8] to a German real-world treatment population.

To facilitate comparison to earlier publications from Hohnloser et al., this analysis was performed using comparable methods and applying the same research database [10, 24]. In a first analysis, Hohnloser et al. described the safety profile of apixaban (*n* = 3,633), dabigatran (*n* = 3,138), rivaroxaban (*n* = 12,063), and phenprocoumon (*n* = 16,179) in NVAF patients newly initiated on anticoagulation in 2013 and 2014 [24]. A subsequent publication analyzed effectiveness and safety of newly initiated phenprocoumon (*n* = 23,823), apixaban (*n* = 10,117), dabigatran (*n* = 5,122), or rivaroxaban (*n* = 22,143) in 61,205 patients with NVAF between 2013 to 2015 [10]. The present study identified fewer patients initiating phenprocoumon (*n* = 13,319) over a four year period (2015 to 2018), probably reflecting the observed decreasing number of phenprocoumon prescriptions in Germany over the study timeframe by approximately 30% according to the German Drug Prescription Reports 2016 vs 2019 representing the German market activity [25, 26].

The present study revealed similar crude event rates in edoxaban initiated patients compared to the rates for apixaban, dabigatran, and rivaroxaban reported by Hohnloser et al. [10, 24]. For phenprocoumon treated patients, slightly lower crude event rates for all effectiveness outcomes except for all-cause mortality were found in this study, whereas for the safety endpoints, the crude event rates of major bleeding, intracranial hemorrhage, and any bleeding were higher for phenprocoumon compared to Hohnloser et al. [10]. On the other hand, gastrointestinal bleeding occurred at similar rates as in the studies of Hohnloser et al. [10, 24]. The small observed differences in crude effectiveness and safety event rates between the studies might be related to patients’ different follow-up duration. Hohnloser et al. [10, 24] used a follow-up period of at least two quarters while our study used a post-index period of at least three quarters based on the published study report from Basic et al. [20]. Thus, the allowed shorter duration of follow-up depicted in Hohnloser et al. [10, 24] compared to our study might contribute to an underestimation of risks and, therefore, to more favorable results for NOACs than reported in our study. Especially for VKAs like phenprocoumon, higher event rates are observed shortly after treatment initiation in clinical practice since patients need to be adjusted regarding dosage individually through regular monitoring of the INR [27]. Since NOAC treatment is not INR-guided [28], described effects after treatment initiation do not appear which again favors the effectiveness and safety profile of NOACs when analyzing a shorter study period. Overall, the frequency of outcome events in our study was homogenous compared to the findings of Hohnloser et al. [10, 24], although our study used a different study drug (edoxaban) for comparison with phenprocoumon. This is reassuring and reflects the robustness of our results in the sense that our study was able to measure similar effects for the comparison of edoxaban vs phenprocoumon, based on similar methods and the same data source used in the studies of Hohnloser et al. [10, 24].

Hohnloser et al. confirmed the effectiveness and safety of NOACs (apixaban, dabigatran, and rivaroxaban) in NVAF patients compared to phenprocoumon, the most widely used VKA in Germany, with all three NOACs showing significantly lower risk when considering HRs of stroke/SE, ischemic stroke, and hemorrhagic stroke compared to phenprocoumon [10]. Extending these observations to edoxaban vs phenprocoumon, the present study revealed lower risks for stroke/SE, all strokes, and hemorrhagic stroke. Similarly, safety endpoints revealed results consistent with Hohnloser et al. [10, 24] demonstrating significantly improved safety with fewer major bleedings, intracranial hemorrhage, and any bleeding in patients initiating edoxaban treatment compared to phenprocoumon.

Overall, the present study demonstrated that edoxaban was generally more effective than phenprocoumon for the prevention of effectiveness and safety events in NVAF patients. These findings are in line with other real-world studies on the comparative effectiveness and safety of edoxaban vs VKA from Paschke et al. and Marston et al. assessed in different German databases [9, 29]. The study by Paschke et al., which was conducted based on a large sample of AF patients (*n* = 837,430), included the highest number of edoxaban patients (*n* = 14,666). Although patients treated with direct oral anticoagulants (dabigatran, apixaban, rivaroxaban, edoxaban) had an overall higher risk for stroke (HR: 1.32, 95% CI: 1.29–1.35) and a lower risk for bleeding (HR: 0.89, 95% CI: 0.88–0.90) compared to phenprocoumon, the risk for stroke (HR: 0.88, 95% CI: 0.74–1.05), and for bleeding (HR: 0.74, 95% CI: 0.68–0.81) was lower for edoxaban when analyzed separately [9]. A similar trend for a superior efficacy and safety of edoxaban over phenprocoumon was also demonstrated in the present work. The study by Marston et al. was based on a sample of AF patients (*n* = 21,038) from an administrative database in Germany, who were treated with VKA or a NOAC. As the study period reached until mid-2017, the study included only a small number of edoxaban patients (*n* = 1,236). In comparison with VKA, adjusted combined risks of ischemic stroke or SE were lower for edoxaban patients (HR: 0.64, 95% CI: 0.60–0.87). In addition, the risk of major bleeding was lower for edoxaban compared to VKA (HR: 0.47, 95% CI: 0.40–0.55) [29]. Although the study by Marston et al. encompassed a smaller sample of edoxaban patients and had a shorter follow-up for edoxaban patients than the present study, these results also indicate a better efficacy and safety profile of edoxaban compared with VKA.

The clinical phase III trial ENGAGE AF-TIMI 48 compared edoxaban to warfarin and showed that edoxaban was at least as effective and safe as the VKA warfarin. Patients from the modified intention-to-treat population treated with edoxaban revealed a 21% reduction in adjusted risk for the primary effectiveness endpoint stroke/SE compared to warfarin [8]. For the primary safety endpoint major bleeding, the pivotal trial revealed a significantly lower risk for edoxaban compared to warfarin treated patients [8], which could be confirmed in the present study for the comparison of edoxaban and phenprocoumon. Overall, the comparison of our study to the clinical trial yielded mostly consistent results for the effectiveness and safety outcome comparison of edoxaban vs VKA warfarin or phenprocoumon. However, the generalizability of results is limited by fundamental disparities between patients participating in clinical trials and patients under real-world conditions who are usually older, have multiple comorbidities and receive more and frequently changing comedications, so that many patients treated in clinical practice will rarely enter clinical trials [11]. Moreover, deviations of findings might be associated with missing power of the clinical trial for subgroups analyses, a too short study period for identification of long-term safety endpoints, and non-consideration of the complexity of real-world clinical decision-making [30, 31]. The comparison to observational studies tackles some of these shortcomings, underlining the scientific merit of our study which demonstrates that the beneficial effects seen in the ENGAGE AF-TIMI 48 can also be achieved in real-world use of edoxaban where large subgroups of patients are of old age, show frailty, and have multiple comorbidities and medications.

In line with other studies on geriatric subgroups [8, 11, 32–34], our analysis yielded homogenous results regarding effectiveness and safety across subgroups according to age, comorbidity burden, presence of frailty, and polypharmacy despite heterogenous patient populations. If observed, effect modifications were likely due to small sample sizes in subgroups. The results indicate that in clinical practice, a balance needs to be struck between preserving the benefits of prevention of thromboembolism and potential bleeding risk in a patient population having numerous comorbidities and concomitant medications. Our findings clearly suggest that the overall benefits of anticoagulation with edoxaban outweigh the risks, even in elderly multimorbid patients. However, associated factors such as renal insufficiency or prescription of drugs interacting with NOAC elimination are well known to be clearly associated with increased bleeding risk. Therefore, the optimal anticoagulation strategy in clinical practice should be individually customized for each patient, considering the patient’s age, body weight, comorbidities, and concomitant medications, as well as the individual risk of thromboembolic and bleeding events, and personal preferences.

### Strength and limitations

The strength of this study is the large and representative database consisting of claims information of approximately 8.8 million insurees, and the data completeness with respect to follow-up and drug prescriptions. Some limitations due to the nature of the underlying data have to be considered when interpretating the results. Analysis of observational data can only establish association between variables but is unable to determine causality. Even though the analysis is adjusted for patient baseline demographic and clinical characteristics, the possibility of residual confounding remains. As claims data are primarily collected for reimbursement purposes and not for purposes of research, certain clinical and laboratory parameters are not covered. For example, the INR could not be considered, even though effectiveness and safety of VKA treatment is highly dependent on the quality of anticoagulation control. Nevertheless, anticoagulation control in Germany seems to be above the international average with a mean time in therapeutic range of 66% and higher [35, 36]. Moreover, analysis of claims data is subject to limitations inherent to potential coding errors and missing data. Among these limitations of administrative data with regard to drug use is that we only observe the prescription and dispensation of drugs. However, the final intake of the medication and thus the compliance of the individual patient cannot be conclusively represented and therefore it is not possible to differentiate between intentional and unintentional pauses. We calculated exposure based pharmacoepidemiological methodology used in similar real-world studies to account for those restrictions [20, 37]. However, the extent of these prementioned aspects may be comparable between exposure groups so that the risk of bias should be low.

## Conclusion

In conclusion, findings from this large real-world study indicate better effectiveness and safety of treatment with edoxaban compared to phenprocoumon in patients with NVAF. Edoxaban treatment revealed a numerically lower risk of stroke/SE and was associated with a significantly lower risk for major bleeding in comparison to phenprocoumon. The comparison of outcomes to previous real-world studies [9, 10, 24, 29] showed largely consistent results and strengthens the confidence in our findings. Importantly, our results confirm the findings of the edoxaban pivotal trial ENGAGE AF-TMI 48 in a German real-world population that included geriatric patients as the most vulnerable subgroup with a high burden of multimorbidity and frailty.

## Supplementary Information

**Additional file 1.** Definition of Baseline Characteristics.

**Additional file 2.** Definition of Effectiveness Endpoints (ICD-10-GM Codes).

**Additional file 3.** Definition of Safety Endpoints (ICD-10-GM and OPS Codes).

## Data Availability

A retrospective claims database analysis was conducted using anonymized claims from the “Institut für angewandte Gesundheitsforschung Berlin” (InGef) Research Database. The data used in this study cannot be made available in the manuscript, the additional files, or in a public repository due to German data protection laws (Bundesdatenschutzgesetz). To facilitate the replication of results, anonymized data used for this study are stored on a secure drive at the InGef. Access to the data used in this study can only be provided to external parties under the conditions of the cooperation contract of this research project and can be assessed upon request after written approval (info@ingef.de), if required.

## Abbreviations

AF: Atrial fibrillation
ASA: Acetylsalicylic acid
ASD: Absolute standardized difference
ATC: Anatomical Therapeutic Chemical Classification System
CCI: Charlson Comorbidity Index
CFI: Claims-based frailty index
CI: Confidence intervals
DESTATIS: German Federal Office of Statistics
EBM: German Physician Fee Schedule
HR: Hazard ratio
ICD-10-GM: International Classification of Diseases, 10^th^ Revision, German Modification
InGef: Institute for Applied Health Research Berlin
INR: International normalized ratio
NOAC: Non-vitamin K oral anticoagulant
NSAID: Non-steroidal anti-inflammatory drug
NVAF: Non-valvular atrial fibrillation
OPS: Key of Operations and Procedures
PZN: German Pharmaceutical Registration Numbers
SD: Standard deviation
SE: Systemic embolism
SHI: Statutory Health Insurance
VKA: Vitamin K antagonist

## Footnotes

1 Apixaban, dabigatran, edoxaban, rivaroxaban, phenprocoumon, or warfarin.

2 C = Congestive heart failure; H = Hypertension, A = Age (≥ 75 years); D = Diabetes mellitus; S = Stroke or transient ischemic attack (TIA) or thromboembolism; V = Vascular diseases; A = Age (65–74 years); Sc = Sex category.

3 H = Hypertension; A = Abnormal renal and liver function; S = Stroke; B = Bleeding; L = Labile International Normalized Ratio (INR); E = Elderly; D = Drugs or alcohol.
