# Supplementary material for: Clinical outcomes in patients receiving edoxaban or phenprocoumon for prevention of stroke in atrial fibrillation: a German real-world cohort study
Source: Thromb J. 2022 Jul 4;20:37. doi: 10.1186/s12959-022-00395-x (PMC9251920; doi:10.1186/s12959-022-00395-x)
Supplement: Supplementary file 1 — Additional file 1. Definition of Baseline Characteristics. [file 12959_2022_395_MOESM1_ESM.docx]

# Additional file 1. Definition of Baseline Characteristics

| **Variable** | **Category** | | **Operational Definition** | |  |
| --- | --- | --- | --- | --- | --- |
| **Patient Demographics** | | | | |  |
| Age | Continuous | | Age was determined in the quarter of the index event for the following age groups: <65, 65-74, 75-84, ≥85 years.  Mean and standard deviation (SD) of age in total and stratified by gender were examined. | |  |
| Gender | Categorical | | Gender was determined in the quarter of the index event and stratified by the following age groups: <65, 65-74, 75-84, ≥85 years. | |  |
| **Healthcare Resource Utilization** | | | | |  |
| All-cause hospitalizations | Continuous | | Assessment of the total number and percentage of eligible patients with all-cause hospitalizations, independently of admission/discharge diagnosis and primary/secondary diagnosis during the individual pre-index period of 365 days before the index date.  Summary measures of all-cause hospitalizations in terms of mean and SD based on the total number of patients were determined. | |  |
| Number of hospital days of all-cause hospitalizations | Continuous | | Assessment of the total number of hospital days of patients with all-cause hospitalizations, independently of admission/discharge diagnosis and primary/secondary diagnosis during the individual pre-index period of 365 days before the index date.  Summary measures of all-cause hospital days in terms of mean and SD based on the total number of patients were determined. | |  |
| All-cause hospitalizations within 30 days before the first dispensation | Continuous | | Assessment of the total number and percentage of eligible patients with all-cause hospitalizations, independently of admission/discharge diagnosis and primary/secondary diagnosis during the individual 30 days before the index date.  Summary measures of all-cause hospitalizations in terms of mean and SD based on the total number of patients were determined. | |  |
| Hospitalizations due to stroke / SE within 30 days before the first dispensation | Continuous | | Assessment of the total number and percentage of eligible patients with hospitalizations with a primary or secondary discharge diagnosis of stroke / SE during the individual 30 days before the index date.  Hospitalizations due to stroke / SE were identified using the ICD-10-GM codes I61.-, I63.-, I64.- and I74.-.  Summary measures of stroke / SE-related hospitalizations in terms of mean and SD based on the total number of patients were determined. | |  |
| Number of outpatient cases | Continuous | | Assessment of the total number and percentage of eligible patients with all-cause outpatient physician visits (based on unique outpatient cases), independently of the reason for the visit during the individual pre-index period of four quarters before the index date.  Summary measures of all-cause outpatient cases in terms of mean and SD based on the total number of patients were determined. | |  |
| Number of unique medications | Continuous | | Assessment of the total number and percentage of eligible patients with prescriptions for pharmaceutical substances (based on unique ATC codes, 7 digits), during the individual pre-index period of 365 days before the index date.  Summary measures of unique pharmaceutical substances in terms of mean and SD based on the total number of patients were determined. | |  |
| **Risk Scores** | | | | | |
| CHA_2_DS_2_-VASc score | Continuous | | The CHA_2_DS_2_-VASc score was calculated for each patient in the individual pre-index period by assigning one point each for hypertension, diabetes mellitus, heart failure, vascular disease (peripheral artery disease, myocardial infarction, aortic plaque), age 65-74 years, female gender, and two points for age ≥75 years, previous stroke or transient ischemic attack (TIA) with a total possible score of nine.^1^  Age and gender were determined in the quarter of the index event.  Ambulatory verified as well as primary and secondary hospital discharge diagnoses within in the four quarters / 365 days before the index date were used to assess the CHA_2_DS_2_-VASc score.  Summary measures of CHA_2_DS_2_-VASc score values in terms of mean and SD based on the total number of patients were assessed. | |  |
| Modified HAS-BLED score | Continuous | | The modified HAS-BLED score was calculated for each patient in the individual pre-index period by assigning one point for the following conditions: hypertension, renal disease, cirrhosis, and stroke, major bleeding event, age 65 and older, use of non-steroidal anti-inflammatory drug, intake of antiplatelet agents, and alcohol abuse with a total possible score of nine after summing up the score across all conditions.^2^  The age was determined in the quarter of the index event.  Ambulatory verified as well as primary and secondary hospital discharge diagnoses within in the four quarters / 365 days before the index date as well as ATC and OPS codes within in the 365 days before the index date were used to assess the modified HAS-BLED score.  Summary measures of modified HAS-BLED score values in terms of mean and SD based on the total number of patients were assessed. | |  |
| Charlson Comorbidity Index | Continuous | | The Charlson Comorbidity Index (CCI) was used to weigh comorbidities in the pre-index period depending on their severity.^3-5^  Ambulatory verified as well as primary and secondary hospital discharge diagnoses within the four quarters / 365 days before the index date were used to calculate the CCI score.  Summary measures of CCI score values in terms of mean and SD based on the total number of patients were assessed. | |  |
| **Comorbidities** | | | | | |
| Ischemic stroke or TIA | Categorical | | Ambulatory verified diagnoses in the four quarters prior to the index quarter as well as primary and secondary hospital discharge diagnoses within the 365 days before the index date were used to assess the number and percentage of patients suffering from ischemic stroke or TIA.  Ischemic stroke or TIA were defined using the ICD-10-GM codes I63.-, I64.-, G45.8, and G45.9. | |  |
| Myocardial infarction | Categorical | | Ambulatory verified diagnoses in the four quarters prior to the index quarter as well as primary and secondary hospital discharge diagnoses within the 365 days before the index date were used to assess the number and percentage of patients suffering from myocardial infarction.  Myocardial infarction was defined using the ICD-10-GM codes I21.- and I22.-. | |  |
| Renal insufficiency | Categorical | | Ambulatory verified diagnoses in the four quarters prior to the index quarter as well as primary and secondary hospital discharge diagnoses within the 365 days before the index date were used to assess the number and percentage of patients suffering from renal insufficiency.  Renal insufficiency was defined using the ICD-10-GM code N18.-. | |  |
| Diabetes mellitus | Categorical | | Ambulatory verified diagnoses in the four quarters prior to the index quarter as well as primary and secondary hospital discharge diagnoses within the 365 days before the index date were used to assess the number and percentage of patients suffering from diabetes mellitus.  Diabetes mellitus was defined using the ICD-10-GM codes E10.-, E11.-, E12.-, E13.-, and E14.-. | |  |
| Hypertension | Categorical | | Ambulatory verified diagnoses in the four quarters prior to the index quarter as well as primary and secondary hospital discharge diagnoses within the 365 days before the index date were used to assess the number and percentage of patients suffering from hypertension.  Hypertension was defined using the ICD-10-GM code I10.-. | |  |
| Congestive heart failure | Categorical | | Ambulatory verified diagnoses in the four quarters prior to the index quarter as well as primary and secondary hospital discharge diagnoses within the 365 days before the index date were used to assess the number and percentage of patients suffering from congestive heart failure.  Congestive heart failure was defined using the ICD-10-GM code I50.-. | |  |
| Coronary heart disease | Categorical | | Ambulatory verified diagnoses in the four quarters prior to the index quarter as well as primary and secondary hospital discharge diagnoses within the 365 days before the index date were used to assess the number and percentage of patients suffering from coronary heart disease.  Coronary heart disease was defined using the ICD-10-GM codes I20.-, I24.-, and I25.-. | |  |
| Any bleeding event | Categorical | | Primary and secondary hospital discharge diagnoses within the 365 days before the index date were used to assess the number and percentage of patients suffering from any bleeding event.  For a complete list of all ICD-10-GM codes and OPS codes which were used to identify any bleeding events please refer to **Table S3**. | |  |
| Major bleeding event | Categorical | | Primary and secondary hospital discharge diagnoses within the 365 days before the index date were used to assess the number and percentage of patients suffering from a major bleeding event.  Major bleeding events were defined as   1. Cases with documented primary and secondary hospital discharge diagnoses of major bleeding event in accordance with ICD-10-GM codes classified as major or intracranial bleeding from **Table S3**.   **OR**   1. Hospital cases in which the:    1. Hospital admission was labelled as emergency admission   **AND**   - 1. An any bleeding (except D62.-) or gastrointestinal bleeding event was coded in accordance with ICD-10-GM codes classified as any or gastrointestinal bleeding from **Table S3** and validated by the documentation of the OPS code 8-800 or the ICD-10-GM code D62.   For a complete list of all ICD-10-GM codes and OPS codes and their operationalization which were used to identify major bleeding events please refer to **Table S3**. | |  |
| **Concomitant Medication** | | | | | |
| Antiplatelet drugs | | Categorical | | Assessment of the number and percentage of patients receiving at least one prescription for antiplatelet medications in the 365 days before or on the index date.  Prescriptions of antiplatelet drugs were identified using ATC code B01AC. | |
| Acetylsalicylic acid | | Categorical | | Assessment of the number and percentage of patients receiving at least one prescription for acetylsalicylic acid (ASA) medications in the 365 days before or on the index date.  Prescriptions of ASA were identified using ATC code B01AC06. | |
| Nonsteroidal anti-inflammatory drugs | | Categorical | | Assessment of the number and percentage of patients receiving at least one prescription for nonsteroidal anti-inflammatory drugs (NSAIDs) in the 365 days before or on the index date.  Prescriptions of NSAIDs were identified using ATC code M01A. | |
| ß-blocker | | Categorical | | Assessment of the number and percentage of patients receiving at least one prescription for ß-blocker in the 365 days before or on the index date.  Prescriptions of ß-blocker were identified using ATC code C07. | |
| Proton-pump inhibitors | | Categorical | | Assessment of the number and percentage of patients receiving at least one prescription for proton-pump inhibitors in the 365 days before or on the index date.  Prescriptions of proton-pump inhibitors were identified using ATC codes A02BC01, A02BC02, A02BC03, A02BC04, A02BC05, A02BC06, A02BC07, A02BC53, and A02BC54. | |

Abbreviations: ASA, acetylsalicylic acid; ATC, Anatomical Therapeutic Chemical Classification System; CCI, Charlson Comorbidity Index; ICD-10-GM, International Classification of Diseases, 10^th^ Revision, German Modification; NSAIDs, nonsteroidal anti-inflammatory drugs; OPS, Key of Operations and Procedures; SD, standard deviation; SE, systemic embolism; TIA, transient ischemic attack

## References

1. Trappe H-J. Atrial Fibrillation. Dtsch Arztebl International 2012;109:1-7

2. Pisters R, Lane DA, Nieuwlaat R, de Vos CB, Crijns HJ, Lip GY. A novel user-friendly score (HAS-BLED) to assess 1-year risk of major bleeding in patients with atrial fibrillation: the Euro Heart Survey. Chest 2010;138:1093-1100

3. Charlson ME, Pompei P, Ales KL, MacKenzie CR. A new method of classifying prognostic comorbidity in longitudinal studies: development and validation. J Chronic Dis 1987;40:373-383

4. Deyo RA, Cherkin DC, Ciol MA. Adapting a clinical comorbidity index for use with ICD-9-CM administrative databases. Journal of clinical epidemiology 1992;45:613-619

5. Quan H, Sundararajan V, Halfon P et al. Coding algorithms for defining comorbidities in ICD-9-CM and ICD-10 administrative data. Med Care 2005;43:1130-1139
