# Supplementary material for: Clinical outcomes in patients receiving edoxaban or phenprocoumon for prevention of stroke in atrial fibrillation: a German real-world cohort study
Source: Thromb J. 2022 Jul 4;20:37. doi: 10.1186/s12959-022-00395-x (PMC9251920; doi:10.1186/s12959-022-00395-x)
Supplement: Supplementary file 2 — Additional file 2. Definition of Effectiveness Endpoints (ICD-10-GM Codes). [file 12959_2022_395_MOESM2_ESM.docx]

# Additional file 2: Definition of Effectiveness Endpoints (ICD-10-GM Codes)

| **ICD-10-GM Code** | **English Description** |
| --- | --- |
| **Stroke/SE** | |
| I61 | Intracerebral hemorrhage |
| I63 | Cerebral infarction |
| I64 | Stroke, not specified as hemorrhage or infarction |
| I74 | Arterial embolism and thrombosis |
| **Stroke** | |
| I61 | Intracerebral hemorrhage |
| I63 | Cerebral infarction |
| I64 | Stroke, not specified as hemorrhage or infarction |
| **Ischemic Stroke** | |
| I63 | Cerebral infarction |
| **Hemorrhagic Stroke** | |
| I61 | Intracerebral hemorrhage |

Abbreviations: ICD-10-GM, International Classification of Diseases, 10^th^ Revision, German Modification; SE, systemic embolism
