# Supplementary material for: Clinical outcomes in patients receiving edoxaban or phenprocoumon for prevention of stroke in atrial fibrillation: a German real-world cohort study
Source: Thromb J. 2022 Jul 4;20:37. doi: 10.1186/s12959-022-00395-x (PMC9251920; doi:10.1186/s12959-022-00395-x)
Supplement: Supplementary file 3 — Additional file 3. Definition of Safety Endpoints (ICD-10-GM and OPS Codes). [file 12959_2022_395_MOESM3_ESM.docx]

# Additional file 3. Definition of Safety Endpoints (ICD-10-GM and OPS Codes)

| **ICD-10-GM / OPS Code** | **Description** | **Bleeding Category** | **Requirement** |
| --- | --- | --- | --- |
| **Major Bleeding** | | | |
| H21.0 | Hyphemia | Major bleeding | None |
| H31.3 | Choroidal hemorrhage and rupture | Major bleeding |  |
| H35.6 | Retinal hemorrhage | Major bleeding |  |
| H43.1 | Vitreous hemorrhage | Major bleeding |  |
| H45.0 | Vitreous hemorrhage in diseases classified elsewhere | Major bleeding |  |
| I31.2 | Hemopericardium, not elsewhere classified | Major bleeding |  |
| I60 | Subarachnoid hemorrhage | Intracranial bleeding |  |
| I61 | Intracerebral hemorrhage | Intracranial bleeding |  |
| I62 | Other nontraumatic intracranial hemorrhage | Intracranial bleeding |  |
| J94.2 | Hemothorax | Major bleeding |  |
| K22.6 | Mallory-Weiss syndrome, Gastro-esophageal laceration-hemorrhage syndrome | Major bleeding |  |
| M25.0 | Hemarthrosis | Major bleeding |  |
| S06.4 | Epidural hemorrhage | Intracranial bleeding |  |
| S06.5 | Traumatic subdural hemorrhage | Intracranial bleeding |  |
| S06.6 | Traumatic subarachnoid hemorrhage | Intracranial bleeding |  |
| S06.8 | Other intracranial injuries: Traumatic hemorrhage: cerebellar, intracranial NOS | Intracranial bleeding |  |
| 8-800 | Transfusion of blood cells: transfusion of whole blood, erythrocyte concentrate and thrombocyte concentrate: whole blood | Any bleeding | Only in combination with an emergency hospital admission and any of the listed ICD-10-GM codes of "Any Bleeding" except D62* |
| D62 | Acute posthemorrhagic anemia | Any bleeding | Only in combination with an emergency hospital admission and any of the listed ICD-10-GM codes of "Any Bleeding" |
| D68.3 | Hemorrhagic disorder due to circulating anticoagulants | Any bleeding | Only in combination with an emergency hospital admission and a documented D62* diagnosis or a blood transfusion (OPS codes 8-800) in the same hospital case |
| D69.8 | Other specified hemorrhagic conditions | Any bleeding |  |
| D69.9 | Hemorrhagic condition, unspecified | Any bleeding |  |
| H11.3 | Conjunctival hemorrhage | Any bleeding |  |
| H92.2 | Otorrhagia | Any bleeding |  |
| I85.0 | Esophageal varices with bleeding | Gastrointestinal bleeding |  |
| I98.3 | Esophageal varices with bleeding in diseases classified elsewhere | Gastrointestinal bleeding |  |
| K22.8 | Other specified diseases of esophagus | Gastrointestinal bleeding |  |
| K25.0 | Gastric ulcer: acute with hemorrhage | Gastrointestinal bleeding |  |
| K25.2 | Gastric ulcer: acute with both hemorrhage and perforation | Gastrointestinal bleeding |  |
| K25.4 | Gastric ulcer: chronic or unspecified with hemorrhage | Gastrointestinal bleeding |  |
| K25.6 | Gastric ulcer: chronic or unspecified with both hemorrhage and perforation | Gastrointestinal bleeding |  |
| K26.0 | Duodenal ulcer: acute with hemorrhage | Gastrointestinal bleeding |  |
| K26.2 | Duodenal ulcer: acute with both hemorrhage and perforation | Gastrointestinal bleeding |  |
| K26.4 | Duodenal ulcer: chronic or unspecified with hemorrhage | Gastrointestinal bleeding |  |
| K26.6 | Duodenal ulcer: chronic or unspecified with both hemorrhage and perforation | Gastrointestinal bleeding |  |
| K27.0 | Peptic ulcer, site unspecified: acute with hemorrhage | Gastrointestinal bleeding |  |
| K27.2 | Peptic ulcer, site unspecified: acute with both hemorrhage and perforation | Gastrointestinal bleeding |  |
| K27.4 | Peptic ulcer, site unspecified: chronic or unspecified with hemorrhage | Gastrointestinal bleeding |  |
| K27.6 | Peptic ulcer, site unspecified: chronic or unspecified with both hemorrhage and perforation | Gastrointestinal bleeding |  |
| K28.0 | Gastrojejunal ulcer: acute with hemorrhage | Gastrointestinal bleeding |  |
| K28.2 | Gastrojejunal ulcer: acute with both hemorrhage and perforation | Gastrointestinal bleeding |  |
| K28.4 | Gastrojejunal ulcer: chronic or unspecified with hemorrhage | Gastrointestinal bleeding |  |
| K28.6 | Gastrojejunal ulcer: chronic or unspecified with both hemorrhage and perforation | Gastrointestinal bleeding |  |
| K29.0 | Acute hemorrhagic gastritis | Gastrointestinal bleeding |  |
| K31.82 | Gastric and duodenal angiodysplasia with bleeding | Gastrointestinal bleeding |  |
| K55.22 | Angiodysplasia of colon with bleeding | Gastrointestinal bleeding |  |
| K57.01 | Diverticulosis of small intestine with perforation and abscess with bleeding | Gastrointestinal bleeding |  |
| K57.03 | Diverticulitis of small intestine with perforation and abscess with bleeding | Gastrointestinal bleeding |  |
| K57.11 | Diverticulosis of small intestine without perforation or abscess with bleeding | Gastrointestinal bleeding |  |
| K57.13 | Diverticulitis of small intestine without perforation or abscess with bleeding | Gastrointestinal bleeding |  |
| K57.21 | Diverticulosis of large intestine with perforation and abscess with bleeding | Gastrointestinal bleeding |  |
| K57.23 | Diverticulitis of large intestine with perforation and abscess with bleeding | Gastrointestinal bleeding |  |
| K57.31 | Diverticulosis of large intestine without perforation or abscess with bleeding | Gastrointestinal bleeding |  |
| K57.33 | Diverticulosis of large intestine without perforation or abscess with bleeding | Gastrointestinal bleeding |  |
| K57.41 | Diverticulitis of both small and large intestine with perforation and abscess with bleeding | Gastrointestinal bleeding |  |
| K57.43 | Diverticulitis of both small and large intestine with perforation and abscess with bleeding | Gastrointestinal bleeding |  |
| K57.51 | Diverticulosis of both small and large intestine without perforation or abscess with bleeding | Gastrointestinal bleeding |  |
| K57.53 | Diverticulitis of both small and large intestine without perforation or abscess with bleeding | Gastrointestinal bleeding |  |
| K57.81 | Diverticulosis of intestine, part unspecified, with perforation and abscess with bleeding | Gastrointestinal bleeding |  |
| K57.83 | Diverticulitis of intestine, part unspecified, with perforation and abscess with bleeding | Gastrointestinal bleeding |  |
| K57.91 | Diverticulosis of intestine, part unspecified, without perforation or abscess with bleeding | Gastrointestinal bleeding |  |
| K57.93 | Diverticulitis of intestine, part unspecified, without perforation or abscess with bleeding | Gastrointestinal bleeding |  |
| K62.5 | Hemorrhage of anus and rectum | Gastrointestinal bleeding |  |
| K66.1 | Hemoperitoneum | Any bleeding |  |
| K92.0 | Hematemesis | Any bleeding |  |
| K92.1 | Melaena | Gastrointestinal bleeding |  |
| K92.2 | Gastrointestinal hemorrhage, unspecified | Gastrointestinal bleeding |  |
| N02 | Recurrent and persistent hematuria | Any bleeding |  |
| N42.1 | Congestion and hemorrhage of prostate | Any bleeding |  |
| N83.6 | Hematosalpinx | Any bleeding |  |
| N85.7 | Hematometra | Any bleeding |  |
| N89.7 | Hematocolpos | Any bleeding |  |
| N93 | Other abnormal uterine or vaginal bleeding | Any bleeding |  |
| N95.0 | Postmenopausal bleeding | Any bleeding |  |
| R04 | Bleeding from the respiratory tract | Any bleeding |  |
| R23.3 | Spontaneous ecchymoses | Any bleeding |  |
| R31 | Unspecified hematuria | Any bleeding |  |
| R58 | Hemorrhage, not elsewhere classified | Any bleeding |  |
| **Intracranial Bleeding** | | | |
| I60 | Subarachnoid hemorrhage | Intracranial bleeding | None |
| I61 | Intracerebral hemorrhage |  |  |
| I62 | Other nontraumatic intracranial hemorrhage |  |  |
| S06.4 | Epidural hemorrhage |  |  |
| S06.5 | Traumatic subdural hemorrhage |  |  |
| S06.6 | Traumatic subarachnoid hemorrhage |  |  |
| S06.8 | Other intracranial injuries: Traumatic hemorrhage: cerebellar, intracranial NOS |  |  |
| **Gastrointestinal Bleeding** | | | |
| I85.0 | Esophageal varices with bleeding | Gastrointestinal bleeding | None |
| I98.3 | Esophageal varices with bleeding in diseases classified elsewhere |  |  |
| K22.8 | Other specified diseases of esophagus |  |  |
| K25.0 | Gastric ulcer: acute with hemorrhage |  |  |
| K25.2 | Gastric ulcer: acute with both hemorrhage and perforation |  |  |
| K25.4 | Gastric ulcer: chronic or unspecified with hemorrhage |  |  |
| K25.6 | Gastric ulcer: chronic or unspecified with both hemorrhage and perforation |  |  |
| K26.0 | Duodenal ulcer: acute with hemorrhage |  |  |
| K26.2 | Duodenal ulcer: acute with both hemorrhage and perforation |  |  |
| K26.4 | Duodenal ulcer: chronic or unspecified with hemorrhage |  |  |
| K26.6 | Duodenal ulcer: chronic or unspecified with both hemorrhage and perforation |  |  |
| K27.0 | Peptic ulcer, site unspecified: acute with hemorrhage |  |  |
| K27.2 | Peptic ulcer, site unspecified: acute with both hemorrhage and perforation |  |  |
| K27.4 | Peptic ulcer, site unspecified: chronic or unspecified with hemorrhage |  |  |
| K27.6 | Peptic ulcer, site unspecified: chronic or unspecified with both hemorrhage and perforation |  |  |
| K28.0 | Gastrojejunal ulcer: acute with hemorrhage |  |  |
| K28.2 | Gastrojejunal ulcer: acute with both hemorrhage and perforation |  |  |
| K28.4 | Gastrojejunal ulcer: chronic or unspecified with hemorrhage |  |  |
| K28.6 | Gastrojejunal ulcer: chronic or unspecified with both hemorrhage and perforation |  |  |
| K29.0 | Acute hemorrhagic gastritis |  |  |
| K31.82 | Gastric and duodenal angiodysplasia with bleeding |  |  |
| K55.22 | Angiodysplasia of colon with bleeding |  |  |
| K57.01 | Diverticulitis of small intestine with perforation and abscess with bleeding |  |  |
| K57.03 | Diverticulitis of small intestine with perforation and abscess with bleeding |  |  |
| K57.11 | Diverticulosis of small intestine without perforation or abscess with bleeding |  |  |
| K57.13 | Diverticulitis of small intestine without perforation or abscess with bleeding |  |  |
| K57.21 | Diverticulitis of large intestine with perforation and abscess with bleeding |  |  |
| K57.23 | Diverticulitis of large intestine with perforation and abscess with bleeding |  |  |
| K57.31 | Diverticulosis of large intestine without perforation or abscess with bleeding |  |  |
| K57.33 | Diverticulitis of large intestine without perforation or abscess with bleeding |  |  |
| K57.41 | Diverticulitis of both small and large intestine with perforation and abscess with bleeding |  |  |
| K57.43 | Diverticulitis of both small and large intestine with perforation and abscess with bleeding |  |  |
| K57.51 | Diverticulosis of both small and large intestine without perforation or abscess with bleeding |  |  |
| K57.53 | Diverticulitis of both small and large intestine without perforation or abscess with bleeding |  |  |
| K57.81 | Diverticulitis of intestine, part unspecified, with perforation and abscess with bleeding |  |  |
| K57.83 | Diverticulitis of intestine, part unspecified, with perforation and abscess with bleeding |  |  |
| K57.91 | Diverticulosis of intestine, part unspecified, without perforation or abscess with bleeding |  |  |
| K57.93 | Diverticulitis of intestine, part unspecified, without perforation or abscess with bleeding |  |  |
| K62.5 | Hemorrhage of anus and rectum |  |  |
| K92.1 | Melaena |  |  |
| K92.2 | Gastrointestinal hemorrhage, unspecified |  |  |
| **Any Bleeding** | | | |
| All codes from above | All major, intracerebral, gastrointestinal, or any bleedings defined above | Any bleedings | None |

Abbreviations: ICD-10-GM, International Classification of Diseases, 10^th^ Revision, German Modification; OPS, Key of Operations and Procedures
